# Gut Microbiome Composition and Dynamics in Hospitalized COVID-19 Patients and Patients with Post-Acute COVID-19 Syndrome

**DOI:** 10.3390/ijms25010567

**Published:** 2024-01-01

**Authors:** Monta Brīvība, Laila Silamiķele, Līga Birzniece, Laura Ansone, Kaspars Megnis, Ivars Silamiķelis, Līva Pelcmane, Daniella Borisova, Maija Rozenberga, Lauma Jagare, Ilze Elbere, Jānis Kloviņš

**Affiliations:** Latvian Biomedical Research and Study Centre, LV-1067 Riga, Latvia; laila.silamikele@biomed.lu.lv (L.S.); liga.birzniece@biomed.lu.lv (L.B.); laura.ansone@biomed.lu.lv (L.A.); kaspars.megnis@biomed.lu.lv (K.M.); ivars.silamikelis@biomed.lu.lv (I.S.); liva.pelcmane@biomed.lu.lv (L.P.); daniella.borisova@biomed.lu.lv (D.B.); ilze.elbere@biomed.lu.lv (I.E.); klovins@biomed.lu.lv (J.K.)

**Keywords:** COVID-19, gut microbiome, post-acute COVID-19 syndrome

## Abstract

The gut microbiome plays a pivotal role in the modulation of host responses during viral infections, and recent studies have underscored its significance in the context of coronavirus disease 2019 (COVID-19). We aimed to investigate the dynamics and compositional changes in the gut microbiome of COVID-19 patients, addressing both the acute phase and the recovery process, with a particular focus on the emergence of post-COVID-19 conditions. Involving 146 COVID-19 patients and 110 healthy controls, this study employed a shotgun metagenomics approach for cross-sectional and longitudinal analyses with one- and three-month follow-ups. We observed a decline in taxonomic diversity among hospitalized COVID-19 patients compared to healthy controls, while a subsequent increase in alpha diversity was shown during the recovery process. A notable contribution of *Enterococcus faecium* was identified in the acute phase of the infection, accompanied by an increasing abundance of butyrate-producing bacteria (e.g., *Roseburia*, *Lachnospiraceae_unclassified*) during the recovery period. We highlighted a protective role of the *Prevotella* genus in the long-term recovery process and suggested a potential significance of population-specificity in the early gut microbiome markers of post-acute COVID-19 syndrome. Our study represents distinctive gut microbiome signatures in COVID-19, with potential diagnostic and prognostic implications, pinpointing potential modulators of the disease progression.

## 1. Introduction

The coronavirus disease (COVID-19) continues to be a major public health concern, characterized by notable morbidity and mortality rates [1]. Additionally, around 50% of patients are not able to fully recover and experience prolonged symptoms persisting for more than three months following the initial infection [2]. Even though COVID-19 is mainly a respiratory illness, around 20–50% of patients report gastrointestinal symptoms, which may be explained by the presence of angiotensin-converting enzyme 2 (ACE2) receptors on the gastrointestinal tract and the ability of the virus to persist in the gut even longer than in the respiratory system [3,4]. Extensive research has already been conducted on the interplay between COVID-19 and gut microbiome, revealing various potential underlying mechanisms. These include the role of gut microbiome in influencing host immunity, the modulation of host responses by microbiome metabolites, and the direct interaction between the microbiome and the ACE2 receptor [5]. Mendelian randomization studies indicate a causal connection between the disease and particular bacteria [6]. However, the observed changes in the gut microbiome of COVID-19 patients may also result from or contribute to the disease. Determining causality becomes even more complex due to the substantial number of COVID-19 patients, especially those in critical condition (up to 75%), who have been administered antibiotics, disrupting the stability of the gut microbiome ecosystem [7].

Several conclusions have already arisen from the extensive research on the modulations in the gut microbiome of COVID-19 patients. Firstly, there is a widely accepted consensus about the significant decline in alpha diversity of the gut microbiome of COVID-19 patients compared to healthy individuals [8]. Moreover, the inverse association has been demonstrated between alpha diversity and disease severity, though the data underlying this hypothesis are conflicting [9,10]. The gut microbiome compositions in individuals with COVID-19 and healthy individuals are entirely distinct according to the beta diversity analyses [11]. Within the taxonomic profiles, there is a significantly higher abundance of beneficial bacteria observed in the gut microbiome of healthy individuals compared to COVID-19 patients (e.g., *Bifidobacterium*, *Faecalibacterium*, and *Roseburium* spp.), while the predominance of *Bacteroides*, *Enterococcus*, *Rothia*, and *Lactobacillus* spp. is observed in COVID-19 patients [12,13]. Moreover, in individuals with severe COVID-19, opportunistic pathogens from the *Enterobacteriaceae* family (such as *Escherichia coli* and *Klebsiella pneumoniae*) and the *Enterococcus* genus (such as *Enterococcus faecalis*) are identified at higher abundance [13]. Finally, the recently reported population specificity in the association between baseline gut microbiome patterns and the severity of COVID-19 adds complexity to concluding the contribution of gut bacteria and suggests the necessity for separate population-level studies [14].

It is estimated that the composition of the gut microbiome restores within six months from the disease onset for patients managing to fully recover from the disease, while for those experiencing post-acute COVID-19 syndrome (long COVID), the characteristic dysbiosis of the gut microbiome may last even for one year since the initial infection. The gut microbiomes of long COVID-19 patients exhibit higher levels of *Ruminococcus gnavus* and *Bacteroides vulgatus*, and lower levels of *Faecalibacterium prausnitzii*. Moreover, there are distinct gut microbiome profiles found in long COVID-19 patients experiencing specifically persistent respiratory and neuropsychiatric symptoms [15]. For example, a notable prevalence of *Veinollella* was detected in COVID-19 patients experiencing respiratory dysfunction during the three-month follow-up [16]. Interestingly, an introduction of fecal material from post-COVID-19 individuals adversely influenced the lungs of recipient mice in the absence of severe acute respiratory syndrome coronavirus 2 (SARS-CoV-2), contributing to a weakening of the host’s defense against bacterial infection [17]. Despite the evident link between long COVID-19 and dysbiosis, there is a limited number of original research articles addressing this topic. In particular, research aimed at identifying alterations in the gut microbiome of long COVID-19 patients at various time points since the disease onset is essential, given its potential clinical relevance for the early identification of risk groups.

In this study, we explore the Latvian population-specific COVID-19-related gut microbiome profiles. We analyze the dynamics of these profiles during the progression of the disease, particularly in severe cases, and aim to identify potential early markers for long COVID-19 within the gut microbiome composition. Employing a shotgun metagenomics approach, we examine stool samples from both the general healthy population and COVID-19 patients, considering long-term clinical manifestations of the infection, with a substantial portion of critically ill patients also involved in longitudinal sampling.

## 2. Results

The study included 146 COVID-19 patients and 110 control subjects, which, together with the longitudinal follow-up samples for the hospitalized patients, comprised 341 stool samples analyzed in total. The characteristics of the study population are summarized in Table 1.

The shotgun metagenomics was the method of choice for the characterization of the composition of the gut microbiome in different subgroups of the study, providing an average of 28.40 ± 11.40 million sequencing reads per sample and ensuring the identification of 321 and 580 unique taxa at genus and species levels, respectively. Five different comparisons for the evaluation of gut microbiome composition were applied considering the disease status, presence of long-term complications, and time of the stool sample collection: (1) hospitalized COVID-19 patients against control individuals (COVID-19 vs. Controls); (2) stool samples collected from the hospitalized patients around 1 month since the COVID-19 acute phase against samples collected during the acute phase of the disease (COVID-1m vs. COVID-acute); (3) stool samples collected from hospitalized patients around 3 months since the COVID-19 acute phase against samples collected from the same individuals during the acute phase (COVID-3m vs. COVID-acute); (4) stool samples collected from the hospitalized patients around 3 months since the COVID-19 acute phase against samples collected around 1 month since the COVID-19 acute phase (COVID-3m vs. COVID-1m); (5) retrospective stool samples from patients developing long COVID-19 phenotype (both hospitalized and ambulatory) against recovered patients (long COVID-19 vs. Recovered). To elucidate the impact of environmental variables on the gut microbiome composition, a canonical-correlation analysis (CCA) coupled with permutational multivariate analysis of variance (PERMANOVA) was performed. The analysis unraveled the significance of several environmental variables such as age (R^2^ = 0.01; *p*-value = 1.20 × 10^−2^), body mass index (BMI) (R^2^ = 0.01; *p*-value = 4.00 × 10^−3^), and antibiotic usage, with the greater importance of the presence of antibiotic therapy, explaining around 2% (R^2^ = 0.02; *p*-value ≤ 1.00 × 10^−3^) of the variability; consequently, all the aforementioned factors were employed as covariates in the subsequent analyses of differential abundance (Figure S1). Since the use of Remdesivir did not appear among the environmental factors significantly shaping the gut microbiota (R^2^ = 0.004; *p*-value = 5.80 × 10^−2^), antiviral therapy was not considered as a significant factor in downstream analyses (Table S1).

To mitigate the inevitable effect of the antibiotic therapy biasing the gut microbiome analysis in the COVID-19 patient cohort, the differential abundance analysis comparing the patients with antibiotic therapy prescribed during the study with patients not administering the antibiotics was conducted, revealing seven antibiotics-related taxa that were excluded from further interpretation within the contrasts of interest (Table S2, Figure S2).

### 2.1. COVID-19-Characteristic Gut Microbiome Composition

**Figure 1:**
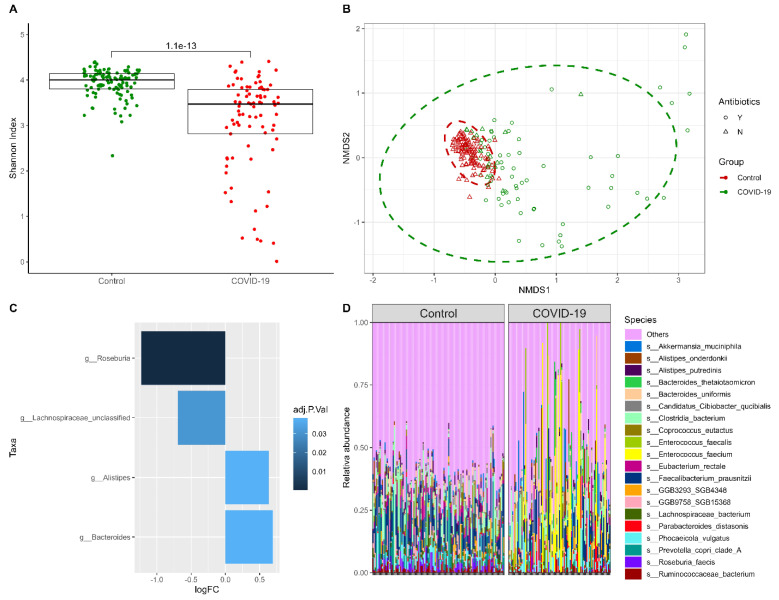
**Alterations in gut microbiome of COVID-19 patients compared to controls.** (**A**) Alpha diversity calculated by the Shannon index. (**B**) Beta diversity characterizing and comparing samples from COVID-19 patients (red) and healthy controls (green). Administration of antibiotics is depicted by the shape of each sample: yes—circles, no—triangles. The non-metric multidimensional scaling (NMDS) plot is based on the Bray–Curtis distance measure. (**C**) Differential abundance at genus and species levels expressed as log2 fold change (LogFC). Positive LogFC represents taxa with increased abundance in the COVID-19 patient group and negative LogFC—in the healthy control group. The intensity of the blue color reflects the false discovery rate (FDR) of the identified associations. (**D**) Taxonomy bar plot depicting 20 of the most representative species of samples in both analyzed groups.

We compared the gut microbiome composition of 92 hospitalized COVID-19 patients with 110 healthy controls reporting no previous infection with SARS-CoV-2 at the time of involvement in the study. The stool samples of the patient group were donated during the acute phase of the disease, and the majority of patients (73%) underwent antibiotic therapy during the participation in the study. The comparison of microbial community variation calculated for each sample (alpha diversities) and quantified as Shannon indexes at the species level revealed a significantly lower alpha diversity in COVID-19 patients compared to healthy controls (median Shannon index in COVID-19 patients = 3.47, interquartile range (IQR) = 2.81–3.79; median in controls = 4.00, IQR = 3.80–4.14; *p*-value = 1.08 × 10^−13^) (Figure 1A). In addition to the alterations in intra-sample diversity, a significant difference in variability in microbial community composition among the analyzed samples (beta-dispersion) between groups was observed (R^2^ = 0.05; *p*-value ≤1.00 × 10^−4^) (Figure 1B,D). Differential abundance analysis was executed at both the species and genus levels, revealing four significantly altered genera in total, with two taxa per each of the groups studied. Bacteria belonging to the *Roseburia* genus exhibited the most prominent alterations, showing reduced levels in the gut microbiome of COVID-19 patients compared to controls (Figure 1C, Table S2). No significant hits were identified at the species level.

### 2.2. Alterations in the Taxonomic Profile of Gut Microbiome in Hospitalized COVID-19 Patients during the Disease Progression

**Figure 2:**
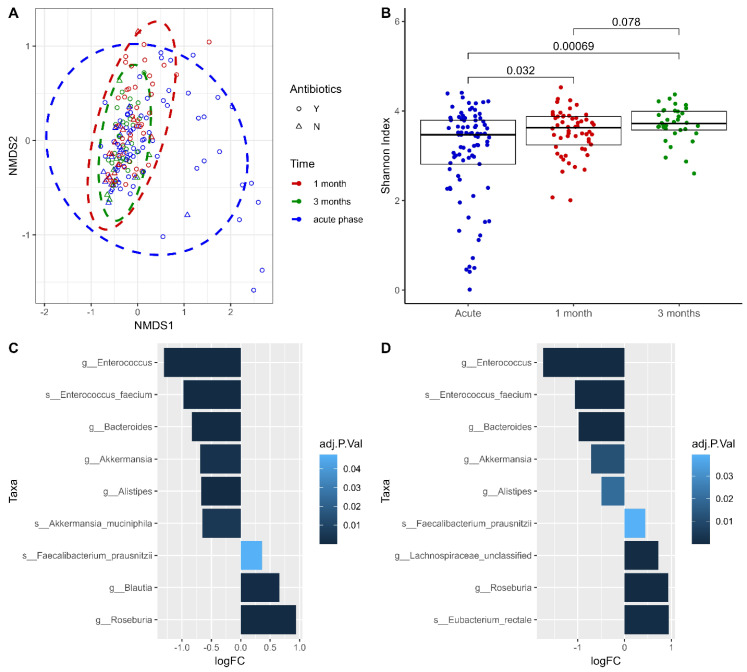
**Alterations of gut microbiome in hospitalized COVID-19 patients during the course of the disease**. (**A**) Beta diversity characterizing and comparing samples collected during the acute phase of the disease (blue) with samples collected around 1 month (red) and around 3 months (green) since the COVID-19 acute phase. Administration of antibiotics is depicted by the shape of each sample: yes—circles, no—triangles. The non-metric multidimensional scaling (NMDS) plot is based on the Bray–Curtis distance measure. (**B**) Alpha diversity calculated by the Shannon index. (**C**) Differential abundance at genus and species levels expressed as log2 fold change (LogFC). Positive LogFC represents taxa with increased abundance in the samples collected after 1 month since the COVID-19 acute phase and negative LogFC—at the acute phase. (**D**) Differential abundance at genus and species levels (expressed as LogFC). Positive LogFC represents taxa with increased abundance in the samples collected after 3 months since the COVID-19 acute phase and negative LogFC—at the acute phase. The intensity of the blue color reflects the false discovery rate (FDR) of the identified associations.

Further comparisons were made longitudinally in hospitalized COVID-19 patients, only comparing the gut microbiome profiles characteristic to the acute phase of the disease, with samples collected around one month (33 ± 5 days) and three months (90 ± 4 days) later. Although a notable and gradual increase in alpha diversity (Shannon indexes) was observed in one-month- (median = 3.62; IQR = 3.24–3.88) and three-month- (median = 3.72; IQR = 3.57–3.99) gut microbiome profiles compared to acute phase (median = 3.47; IQR = 2.81–3.79), only the comparisons of COVID-1m vs. COVID-acute (*p*-value = 3.20 × 10^−2^) and COVID-3m vs. COVID-acute (*p*-value = 6.90 × 10^−4^) reached statistical significance (Figure 2B). In addition, a distinct beta-diversity clustering was observed among the samples collected in three distinct time points according to the PERMANOVA (R^2^ = 0.02; *p*-value = <1.00 × 10^−4^), with notably higher dispersion in samples of the acute phase (Figure 2A).

The comparison of COVID-1m vs. COVID-acute in the differential abundance analysis revealed six genera and three species of altered relative abundance. Out of them, six differentially abundant taxa expressed significantly reduced levels in one-month samples compared to the relative abundances observed during the acute phase of the infection. The most notable changes in abundance were observed in *Enterococcus* at the genus level (log2 fold change (logFC) = −1.32; false discovery rate (FDR) = 3.50 × 10^−7^) and, more specifically, *Enterococcus faecium* at the species level (logFC = −0.98; FDR = 1.31 × 10^−5^) both exhibiting a significant reduction one month after the acute phase. A contrasting trend was noted for *Roseburia*, displaying a significant increase (logFC = 0.94; FDR = 2.16 × 10^−6^) over one month (Figure 2C, Table S2).

The gut microbiome profiles of samples collected around three months after the acute phase showed significant alterations in levels of nine taxa in total, six of them at the genus level and three at the species level. The same previously highlighted taxa (*Enterococcus:* logFC = −1.74, FDR = 6.58 × 10^−9^; *Enterococcus faecium:* logFC = −1.06, FDR = 7.91 × 10^−5^) demonstrated the most prominent changes in the relative abundances with an even stronger effect than observed in the COVID-1m vs. COVID-acute contrast before. Consistently, *Roseburia* (logFC = 0.94; FDR = 5.78 × 10^−5^) showed elevated levels even after three months, alongside the *Faecalibacterium prausnitzii* (logFC = 0.44; FDR = 3.94 × 10^−2^) (Figure 2D). The overlap between the top hits deriving from the contrasts COVID-1m vs. COVID-acute and COVID-3m vs. COVID-acute consisted of seven taxa (Figures S3 and S4).

The only significant result of the comparison between COVID-3m and COVID-1m was *Eubacterium rectale* (logFC = 0.98; FDR = 1.21 × 10^−3^), which also appeared in the contrast of COVID-3m vs. COVID-1m, though was significantly affected by the antibiotic therapy (logFC = 0.94; FDR = 4.43 × 10^−2^) (Table S2, Figure S2).

### 2.3. Early Alterations in the Gut Microbiome Composition of Patients Experiencing Long-Term Complications

**Figure 3:**
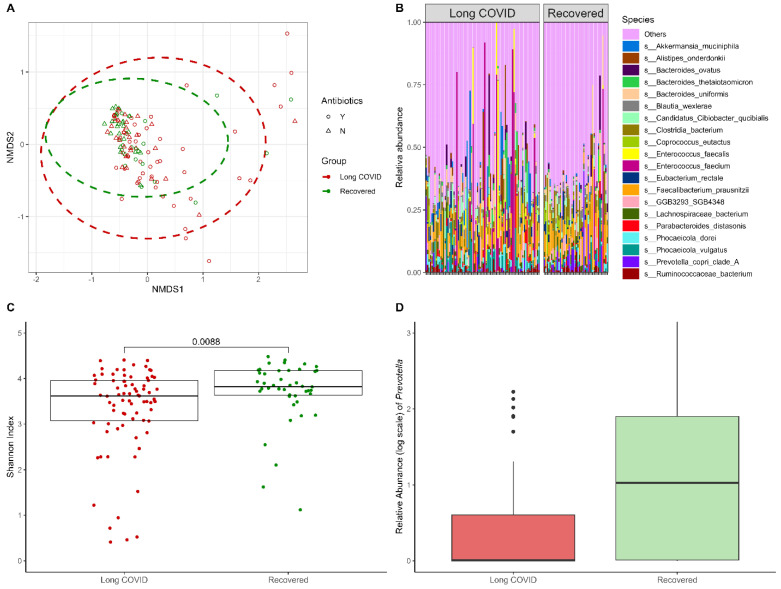
Differences in the gut microbiome close to the acute phase comparing patients later experiencing long-term complications versus patients characterized as fully recovered. (**A**) Beta diversity characterizing and comparing samples collected from COVID-19 patients with long COVID-19 (red)) and from recovered COVID-19 patients (green). Administration of antibiotics is depicted by the shape of each sample: yes—circles, no—triangles. The non-metric multidimensional scaling (NMDS) plot is based on the Bray–Curtis distance measure. (**B**) Taxonomy bar plot depicting 20 of the most representative species of samples in both analyzed groups. (**C**) Alpha diversity calculated by the Shannon index. (**D**) Relative abundance of *Prevotella* spp. in long COVID-19 (red) and recovered (green) patient groups. Boxplots present the median, 25th, and 75th percentiles and, if applicable, outliers.

To pinpoint the early markers of long-term COVID-19-related complications within the composition of the gut microbiome, the metagenomic dataset of 122 stool samples collected from COVID-19 patients close to the acute phase of the disease (around 20 days after disease onset according to the medical records) was used. Among these 122 phenotypically well-characterized patients, 78 corresponded to long COVID-19 phenotype, while 44 were considered as fully recovered. The comparison of Shannon indexes revealed statistically significantly (*p*-value = 8.78 × 10^−3^) lower alpha diversities in patients later experiencing long COVID-related clinical manifestations (median = 3.62; interquartile range (IQR) = 3.07–3.96) compared to fully recovered patients (median = 3.82; IQR = 3.63–4.17) (Figure 3C). No significant differences were observed in beta diversities among the groups of interest (R^2^ = 0.01; *p*-value = 1.42 × 10^−1^) (Figure 3A). The analysis of differential abundance did not reveal any significantly altered taxa at the species level. However, an increased abundance of *Prevotella* spp. was observed at the genus level in stool samples collected from patients who later fully recovered from the disease, as opposed to long COVID-19 patients (logFC = −1.04; FDR = 3.50 × 10^−2^) (Figure 3B,D) (Table S2).

## 3. Discussion

In line with other research studies, our findings reveal an altered gut microbiome profile in COVID-19 patients, showing compositional shifts during both the acute phase and recovery process. Previous research [18] has shown that patients with COVID-19 experience decreased gut microbiome diversity, associated with pro-inflammatory response and an elevated susceptibility to opportunistic infections. This is fully consistent with our results, as we observed significantly lower alpha diversity in COVID-19 patients.

Corresponding to our results, another similarly sized and designed study using a shotgun metagenomic sequencing approach has shown an increase in the relative abundance of different species of the *Bacteroides* genus in the gut microbiome of COVID-19 patients [19]. Furthermore, we observed a decrease in the genus both in COVID-1m and COVID-3m samples compared to the COVID-acute ones; however, Yeoh and colleagues did not detect such an effect in their analysis of recovered patient samples. This could be explained by the relatively short follow-up period of up to 30 days in their study, as our results showed a stronger increase in the contrast using COVID-3m samples. In turn, another case-control study has shown a decrease in the relative abundance of two species of the genus—*B. caccae* and *B. coprophilus* [20]. It should be noted that we observed significant differences in the *Bacteroides* relative abundance depending on antibiotic use; therefore, this might potentially cause discrepancies between the results of different studies. *Alistipes* has been detected as another genus enriched in the feces of COVID-19 patients and, together with *Bacteroides*, increased during the recovery from COVID-19.

In the longitudinal analysis, the strongest depletion was observed in the relative abundance of the *Enterococcus* genus. *Enterococcus* has been associated with COVID-19 in several studies [7,21] and is considered an opportunistic pathogen [22]. An increase in *Enterococcus* has been shown in patients with severe COVID-19 [23]. The increased prevalence of opportunistic pathogens like *Enterococcus* during COVID-19 has been previously attributed to the activated host immune responses triggered by SARS-CoV-2. This involves the activation of pattern recognition receptors, resulting in impaired gut permeability and a disturbance in the equilibrium of the gut microbiome [24]. A previous study has reported an increased relative abundance of *Enterococcus faecalis* in recovered patients who have received antibiotics, while in recovered patients without antibiotic treatment, no changes in the relative abundance were detected [19]. Contrary to prior reports of antibiotic-induced alterations in the gut microbiota favoring opportunistic pathogens, including *Enterococcus*, especially in critically ill COVID-19 patients [7], our study did not detect a difference in the abundance of *Enterococcus* when comparing stool samples from antibiotic users to non-users (Table S2, Figure S2).

*Eubacterium rectale*, *Roseburia*, *Lachnospiraceae_unclassified*, and, to a lesser extent, *Faecalibacterium prausnitzii*, all butyrate producers, were significantly increased in COVID-3m samples compared to the COVID-acute ones. Interestingly, a previous study has shown a significant depletion of *F. prausnitzii* and *E. rectale* in COVID-19 patients compared to controls [25], thus supporting our results. In addition to a significant increase during the recovery, in our study, the butyrate-producing *Roseburia* and *Lachnospiraceae_unclassified* also showed a lower abundance in COVID-19 patient samples compared to healthy controls. This is in complete agreement with a report comparing patients with different levels of COVID-19 severity, where the levels of butyrate-producing bacteria, including *Faecalibacterium prausnitzii*, *Clostridium butyricum*, *Clostridium leptum*, and *Eubacterium rectale*, were notably reduced in critically ill COVID-19 patients in contrast to the general group [13]. A different study has reported depletion of *Blautia*, another butyrate producer, in COVID-19 patients’ feces [20], the relative abundance of which was increased in COVID-1m patients compared to COVID-acute ones in the present study. Butyrate has a crucial role in preventing the overgrowth of opportunistic pathogens, sustaining the integrity of the intestinal mucosal barrier, stimulating the adaptive immune response, bolstering antiviral immunity, and even regulating the expression of angiotensin-converting enzyme 2, crucial for the SARS-CoV-2 entry in the cell [13,26,27,28]. While the impact of altered short-chain fatty acids levels on host cell functions is well-documented, only a few hypotheses have been proposed, explaining the possible mechanisms contributing to the depletion of butyrate-producing bacteria in the gut during the SARS-CoV-2 infection. These include viral infection-induced gut dysbiosis itself [29], along with potential nutrient deficiencies in the host cells [30] as the primary causes of the reduction of butyrate-producing bacteria. Finally, since our data also reveal a significant decline of acetate and propionate-producing *Akkermansia* [31], we may conclude that there is an ongoing shift of short-chain fatty acid-producing bacteria during the recovery process of COVID-19. Further studies involving fecal metabolomics should be conducted to clarify the underlying mechanisms of the particular association.

Conversely, there is a previously reported significant increase in the abundance of common opportunistic pathogens, such as *Enterobacteriaceae* and *Enterococcus*, in individuals with severe COVID-19 compared to the general group [13]. Our results further support this shift in the bacterial community, indicating that COVID-19 severity is associated with an increase in opportunistic pathogens, including *Enterococcus*, and a decrease in the relative abundance of butyrate-producing genera, which is reverted during the recovery from the infection.

Analysis of long COVID-19 vs. Recovered patients revealed a significant enrichment of *Prevotella* spp. in the microbiome of recovered patients. Lu et al. have shown that the abundance of *Prevotella* in the oropharyngeal microbiome positively correlates with the level of C-reactive protein; therefore, *Prevotella* has been suggested as a biomarker in host immune response assessment in COVID-19 patients [32]. Furthermore, in a study comparing a 3-month follow-up group of COVID-19 patients to a mild COVID-19 group and healthy controls, a significant decrease in the abundance of *Prevotella* was shown in the follow-up group [33]. In addition, the overexpression of *Prevotella* proteins is involved in augmenting the severity of the disease [34]. Our results suggest that a higher relative abundance of *Prevotella* in the baseline gut microbiome contributes to a better recovery from COVID-19. Despite earlier associations of *Prevotella* with a negative prognosis, our observations reveal a protective role for this specific genera. This emphasizes the need to investigate the particular interaction at the species and strain levels. Furthermore, these findings may support the hypothesis of population specificity, particularly in identifying early markers of long COVID-19 manifestations.

Several limitations in this study could be addressed by future research. Firstly, the gut microbiome composition among COVID-19 patients was significantly influenced by the use of antibiotic therapy during the disease course. Despite this impact, antibiotic therapy was not considered an exclusion criterion. This decision was made due to the relatively high global incidence of bacterial co-infections among COVID-19 patients, making it challenging to avoid bias related to antibiotics without significantly reducing the sample size [35]. However, we addressed this issue by (1) incorporating the use of antibiotics as covariates in the differential abundance analysis and (2) identifying antibiotic-related effects by conducting a differential abundance analysis comparing antibiotic users to non-users. Taxa discovered in this analysis were excluded from further disease-associated interpretations within the contrasts of interest. Secondly, while all hospitalized patients were considered acute during the collection of the first sample according to their medical reports, the time of stool sample collection relative to the disease onset varied in ambulatory patients, which, together with hospitalized patients, were analyzed in the contrast of long COVID-19 patients versus recovered patients. This heterogeneity limits the ability to confidently conclude about the exact time when the early gut microbiome profiles related to long COVID-19 might be identifiable. Finally, high phenotypic heterogeneity was observed between COVID-19 patients and controls as well as within the COVID-19 patient group; therefore, one may consider choosing matching strategies for the study design and expanding the sample size to find more robust observations.

In summary, the present study provides a complex metagenomics dataset of cross-sectionally and longitudinally collected stool samples reflecting the gut microbiome signatures of COVID-19 during various stages of the virus infection. Our findings align with earlier observations of distinct populations showing significant contributions of previously reported taxa (e.g., *Enterococcus*) and indicate that following SARS-CoV-2 infection, the composition of the gut microbiome tends to shift towards a universal profile. Moreover, we show a significant increase in the beneficial butyrate-producing bacteria during the three-month recovery process, even in critically ill patients. Contrary to previous reports, we emphasize the potential protective role of the *Prevotella* genus in the long-term recovery process. This finding supports the hypothesis of population-specificity [8] when considering gut microbiome profiles for predicting the disease outcome. In conclusion, we believe that our data pinpoint potential diagnostic and prognostic biomarkers for COVID-19 and may be used to develop microbiome-based treatment strategies.

## 4. Methods

### 4.1. Study Design and Sample Collection

In total, 146 patients with a clinically confirmed diagnosis of COVID-19 were enrolled in the study from May 2020 to January 2021. Out of them, 54 patients received outpatient care, and 92 patients were hospitalized during the course of the disease. Within the subgroup of 92 hospitalized patients, 59 individuals participated in the first follow-up conducted after around one month (mean 33 ± 4 days) since the initial patient recruitment, and 36 individuals agreed on the second follow-up visit organized around three months (90 ± 4 days) since the beginning of the study. Each visit comprised both the stool sample collection and questionnaire on the self-assessment of health condition. No follow-ups were conducted for the control subjects and ambulatory patients.

Patients (1) reporting persisting complications for at least 12 weeks and/or (2) having the U09.9 diagnosis code (ICD-10) in their medical records and/or matching the criteria described by Zang et al., 2023 [36] were included in the long COVID-19 subgroup of the study (n = 78). Meanwhile, for 44 patients, the questionnaire and medical record data indicated full recovery; therefore, these were classified as recovered.

For the control group, stool samples from 110 individuals were selected from the Genome Database of the Latvian population (LGDB) collected from individuals of the general population recruited in the LGDB from November 2020 to September 2021, with no SARS-CoV-2 infection detected at the time of sample donation.

Hospitalized patients were recruited during the acute phase of the infection in collaboration with the Riga East University Hospital, Vidzeme Hospital, Liepāja Regional Hospital, and LGDB [37]. Recruitment of ambulatory patients and healthy controls was conducted through private clinical laboratories (E. Gulbja Laboratorija, Ltd., Riga Latvia and Centrālā Laboratorija, Ltd., Riga, Latvia). Written informed consent was obtained from each study participant, and the study protocol was approved by the Central Medical Ethics Committee of Latvia (No. 01-29.1.2/928). The study was conducted following the Code of Ethics of the World Medical Association (Declaration of Helsinki) and The Convention for the Protection of Human Rights and Dignity of the Human Being with regard to the Application of Biology and Medicine: Convention on Human Rights and Biomedicine.

The first stool samples from the hospitalized patients were collected around 8 days after the onset of the disease, while for ambulatory patients, this was performed 30 days after the onset of the disease (ambulatory patient samples were included in the long COVID-19 analysis only). The date of disease onset may potentially lack precision and be unavailable for certain individuals within the cohort, possibly attributed to gaps in healthcare system performance noted early in the pandemic. The follow-up samples were collected only from the hospitalized patients: (1) after around one month (mean 33 ± 4 days) since the initial sample, and (2) around three months (90 ± 4 days) since the initial sampling. Stool samples were collected by the study participants in two aliquots using sterile collection tubes with no buffer added. Each patient reported a precise sample collection date and time by marking it on the tube. Samples were delivered to LGDB within 24 h and stored at −80 °C until further processing. The anthropometric measures were obtained in the form of questionnaires according to the standard procedures of LGDB [37], while the clinical data involving information about the symptomatics were collected from hospital case records.

### 4.2. Sample Processing and Next-Generation Sequencing

Microbial DNA extraction from stool samples was performed using the MGISP-960 Automated Sample Preparation System (MGI Tech Co., Ltd., Wuhan, China) and MagPure Stool DNA LQ Kit (Angen Biotech Co., Ltd., Guangzhou, China). MGIEasy Universal DNA Library Prep Set (MGI Tech Co., Ltd., Wuhan, China) was used for DNA library preparation according to the manufacturer’s instructions, and the following sequencing was done with the DNBSEQ-G400RS sequencing platform using DNBSEQ-G400RS High-throughput Sequencing Set (PE 150) (MGI Tech Co., Ltd., Wuhan, China), providing 150 bp paired-end sequencing reads. Quantity and quality of DNA were evaluated using the Qubit 2.0 fluorometer (ThermoFisher Scientific, Waltham, MA, USA) and Agilent 2100 Bioanalyzer system (Agilent Technologies, Santa Clara, CA, USA), respectively.

### 4.3. Data Analysis

For the raw sequencing reads, adapter clipping and read trimming were performed with fastp 0.20.0. Reads shorter than 100 bp were removed. Host read removal was performed by aligning reads with bowtie2 (version 2.3.5.1) against the GRCh38 (Ensembl release 108) reference genome. Taxonomic assignment was performed with Metaphlan (v4.0.1). The Shannon index at the species level was considered as the alpha diversity measure calculated by the diversity function in the vegan package (v2.6-4) of R (v.4.3.0). The p-values for the comparisons of Shannon indexes were calculated using the Wilcoxon rank sum test. For the analysis of beta diversity, non-metric multidimensional scaling (NMDS) using Bray–Curtis distances was applied within the vegan package (v2.6-4) of R (v.4.3.0). The Permutational Multivariate Analysis of Variance Using Distance Matrices (PERMANOVA) test (adonis2 function) was used for the identification of significant contributing variables together with Canonical Correspondence Analysis (CCA), both implemented in the vegan package (v2.6-4) of R (v.4.3.0). The alterations in taxonomic profiles between different groups of interest were evaluated by R packages edgeR (v3.42.4) limma (v3.56.2) using voom transformation with sample-specific quality weights, adjusting for age, sex, body mass index (BMI), and use of antibiotics. To provide robust results, only the taxa present in at least 10% of samples were included in the differential abundance analysis, and the filterByExpr() function was applied, taking into account the library sizes and the experimental design. The false discovery rate (FDR) threshold <0.05 was set for the identification of significant hits. All of the visualizations were developed in the *ggplot2* package implemented in R (v4.3.0).

The differentially abundant taxa within the gut microbiome were determined in five different contrasts at both genus and species levels depending on the patient disease status, presence of long-term complications, and time of stool sample collection: (1) hospitalized COVID-19 patients against control individuals (COVID-19 vs. Controls); (2) stool samples collected from the hospitalized patients around 1 month since the COVID-19 acute phase against samples collected during the acute phase of the disease (COVID-1m vs. COVID-acute); (3) stool samples collected from hospitalized patients around 3 months since the COVID-19 acute phase against samples collected from the same individuals during the acute phase (COVID-3m vs. COVID-acute); (4) stool samples collected from the hospitalized patients around 3 months since the COVID-19 acute phase against samples collected around 1 month since the COVID-19 acute phase (COVID-3m vs. COVID-1m); (5) retrospective stool samples from patients developing long COVID-19 phenotype (both hospitalized and ambulatory) against recovered patients (long COVID-19 vs. Recovered). Due to missing phenotype data of long-term complications for the part of the study subjects, the analyses were conducted on two separate datasets: (1) metagenomic data of 92 hospitalized patients together with 110 control patients were used for the contrasts: COVID-19 vs. Controls, COVID-1m vs. COVID-acute, COVID-3m vs. COVID-acute, COVID-3m vs. COVID-1m, and (2) metagenomic data from 68 hospitalized patients and 54 ambulatory patients were used for the contrast of long COVID-19 vs. Recovered.

## Figures and Tables

**Table 1:** Characteristics of the study population.

Characteristic	Healthy Individuals (n = 110)	Patients (n = 146)	*p*-Value
Males/females, n (%)	33 (30)/77 (70)	86 (58.90)/60 (41.10)	
Age (years), mean ± SD	36.61 ± 9.43	53.31 ± 15.88	<0.001
BMI (kg/m^2^), mean ± SD	23.20 ± 2.56	27.89 ± 6.12	<0.001
Use of antibiotics, n (%)	0 (0)	83 (56.85)	
Use of antivirals (*Remdesivir*), n (%)	0 (0)	25 (17.12)	
Ambulatory/hospitalized, n (%)	N/A	57 (39.04)/89 (60.96)	
Long COVID/recovered, n (%)	N/A	78 (53.42)/44 (30.14)	
No information on long-term complications, n (%)	N/A	15 (10.27)	

SD—standard deviation; BMI—body mass index; N/A—not applicable.

## Data Availability

The data presented in this study are openly available in the European Nucleotide Archive repository, accession number: PRJEB70508.

## Supplementary Materials

The supporting information can be downloaded at: https://www.mdpi.com/article/10.3390/ijms25010567/s1.

## References

[1] Cortés J., Aguiar P.M.V., Ferrinho P. (2023). COVID-19-Related Adolescent Mortality and Morbidity in Nineteen European Countries. Eur. J. Pediatr..

[2] Woodrow M., Carey C., Ziauddeen N., Thomas R., Akrami A., Lutje V., Greenwood D.C., Alwan N.A. (2023). Systematic Review of the Prevalence of Long COVID. Open Forum Infect. Dis..

[3] Lin L., Jiang X., Zhang Z., Huang S., Zhang Z., Fang Z., Gu Z., Gao L., Shi H., Mai L. (2020). Gastrointestinal symptoms of 95 cases with SARS-CoV-2 infection. Gut.

[4] Gupta A., Madhavan M.V., Sehgal K., Nair N., Mahajan S., Sehrawat T.S., Bikdeli B., Ahluwalia N., Ausiello J.C., Wan E.Y. (2020). Extrapulmonary manifestations of COVID-19. Nat. Med..

[5] Wang H., Xue X., Zhang S. (2022). Microbiome and COVID-19: Long-Term and Complex Influencing Factors. Front. Microbiol..

[6] Song J., Wu Y., Yin X., Ma H., Zhang J. (2023). The Causal Links between Gut Microbiome and COVID-19: A Mendelian Randomization Study. J. Med. Virol..

[7] Righi E., Lambertenghi L., Gorska A., Sciammarella C., Ivaldi F., Mirandola M., Sartor A., Tacconelli E. (2022). Impact of COVID-19 and Antibiotic Treatments on Gut Microbiome: A Role for *Enterococcus* spp.. Biomedicines.

[8] Krasaewes K., Chaiwarith R., Chattipakorn N., Chattipakorn S.C. (2023). Profiles of Gut Microbiome Associated with Clinical Outcomes in Patients with Different Stages of SARS-CoV-2 Infection. Life Sci..

[9] Talukdar D., Bandopadhyay P., Ray Y., Paul S.R., Sarif J., D’rozario R., Lahiri A., Das S., Bhowmick D., Chatterjee S. (2023). Association of Gut Microbial Dysbiosis with Disease Severity, Response to Therapy and Disease Outcomes in Indian Patients with COVID-19. Gut Pathog..

[10] Nobre J.G., Delgadinho M., Silva C., Mendes J., Mateus V., Ribeiro E., Costa D.A., Lopes M., Pedroso A.I., Trigueiros F. (2022). Gut Microbiome Profile of COVID-19 Patients: Prognosis and Risk Stratification (MicroCOVID-19 Study). Front. Microbiol..

[11] Maddah R., Goodarzi V., Asadi-Yousefabad S.L., Abbasluo M., Shariati P., Shafiei Kafraj A. (2023). Evaluation of the Gut Microbiome Associated with COVID-19. Inform. Med. Unlocked.

[12] Hazan S., Stollman N., Bozkurt H.S., Dave S., Papoutsis A.J., Daniels J., Barrows B.D., Quigley E.M., Borody T.J. (2022). Lost Microbes of COVID-19: Bifidobacterium, Faecalibacterium Depletion and Decreased Microbiome Diversity Associated with SARS-CoV-2 Infection Severity. BMJ Open Gastroenterol..

[13] Tang L., Gu S., Gong Y., Li B., Lu H., Li Q., Zhang R., Gao X., Wu Z., Zhang J. (2020). Clinical Significance of the Correlation between Changes in the Major Intestinal Bacteria Species and COVID-19 Severity. Engineering.

[14] Lymberopoulos E., Gentili G.I., Budhdeo S., Sharma N. (2022). COVID-19 Severity Is Associated with Population-Level Gut Microbiome Variations. Front. Cell. Infect. Microbiol..

[15] Liu Q., Mak J.W.Y., Su Q., Yeoh Y.K., Lui G.C.-Y., Ng S.S.S., Zhang F., Li A.Y.L., Lu W., Hui D.S.-C. (2022). Gut Microbiome Dynamics in a Prospective Cohort of Patients with Post-Acute COVID-19 Syndrome. Gut.

[16] Vestad B., Ueland T., Lerum T.V., Dahl T.B., Holm K., Barratt-Due A., Kåsine T., Dyrhol-Riise A.M., Stiksrud B., Tonby K. (2022). Respiratory Dysfunction Three Months after Severe COVID-19 Is Associated with Gut Microbiome Alterations. J. Intern. Med..

[17] de Almeida V.M., Engel D.F., Ricci M.F., Cruz C.S., Lopes S., Alves D.A., Auriol M.D., Magalhães J., Machado E.C., Rocha V.M. (2023). Gut Microbiome from Patients with COVID-19 Cause Alterations in Mice That Resemble Post-COVID Symptoms. Gut Microbes.

[18] Farsi Y., Tahvildari A., Arbabi M., Vazife F., Sechi L.A., Bonjar A.H.S., Jamshidi P., Nasiri M.J., Mirsaeidi M. (2022). Diagnostic, Prognostic, and Therapeutic Roles of Gut Microbiome in COVID-19: A Comprehensive Systematic Review. Front. Cell. Infect. Microbiol..

[19] Yeoh Y.K., Zuo T., Lui G.C.-Y., Zhang F., Liu Q., Li A.Y., Chung A.C., Cheung C.P., Tso E.Y., Fung K.S. (2021). Gut Microbiome Composition Reflects Disease Severity and Dysfunctional Immune Responses in Patients with COVID-19. Gut.

[20] Wu Y., Cheng X., Jiang G., Tang H., Ming S., Tang L., Lu J., Guo C., Shan H., Huang X. (2021). Altered Oral and Gut Microbiome and Its Association with SARS-CoV-2 Viral Load in COVID-19 Patients during Hospitalization. NPJ Biofilms Microbiomes.

[21] Wu C., Xu Q., Cao Z., Pan D., Zhu Y., Wang S., Liu D., Song Z., Jiang W., Ruan Y. (2021). The Volatile and Heterogeneous Gut Microbiome Shifts of COVID-19 Patients over the Course of a Probiotics-assisted Therapy. Clin. Transl. Med..

[22] Zuo T., Wu X., Wen W., Lan P. (2021). Gut Microbiome Alterations in COVID-19. Genom. Proteom. Bioinform..

[23] Gaibani P., D’Amico F., Bartoletti M., Lombardo D., Rampelli S., Fornaro G., Coladonato S., Siniscalchi A., Re M.C., Viale P. (2021). The Gut Microbiome of Critically Ill Patients With COVID-19. Front. Cell. Infect. Microbiol..

[24] Zhang F., Lau R.I., Liu Q., Su Q., Chan F.K.L., Ng S.C. (2022). Gut microbiota in COVID-19: Key microbial changes, potential mechanisms, and clinical applications. Nat. Rev. Gastroenterol. Hepatol..

[25] Zuo T., Zhang F., Lui G.C.Y., Yeoh Y.K., Li A.Y.L., Zhan H., Wan Y., Chung A.C.K., Cheung C.P., Chen N. (2020). Alterations in Gut Microbiome of Patients With COVID-19 during Time of Hospitalization. Gastroenterology.

[26] Chen J., Vitetta L. (2020). The Role of Butyrate in Attenuating Pathobiont-Induced Hyperinflammation. Immune Netw..

[27] Hodgkinson K., El Abbar F., Dobranowski P., Manoogian J., Butcher J., Figeys D., Mack D., Stintzi A. (2023). Butyrate’s Role in Human Health and the Current Progress towards Its Clinical Application to Treat Gastrointestinal Disease. Clin. Nutr..

[28] Paparo L., Maglio M.A., Cortese M., Bruno C., Capasso M., Punzo E., Ferrucci V., Lasorsa V.A., Viscardi M., Fusco G. (2022). A new butyrate releaser exerts a protective action against SARS-CoV-2 infection in the human intestine. Molecules.

[29] Howell M.C., Green R., McGill A.R., Dutta R., Mohapatra S., Mohapatra S.S. (2021). SARS-CoV-2-induced gut microbiome dysbiosis: Implications for colorectal cancer. Cancers.

[30] Zhou T., Wu J., Zeng Y., Li J., Yan J., Meng W., Han H., Feng F., He J., Zhao S. (2022). SARS-CoV-2 triggered oxidative stress and abnormal energy metabolism in gut microbiota. MedComm.

[31] Lukovac S., Belzer C., Pellis L., Keijser B.J., de Vos W.M., Montijn R.C., Roeselers G. (2014). Differential modulation by Akkermansia muciniphila and Faecalibacterium prausnitzii of host peripheral lipid metabolism and histone acetylation in mouse gut organoids. mBio.

[32] Lu S., Zhou Y., Hu Y., Wang J., Li H., Lin Y., Wang D., Xian J., Zhao S., Ma J. (2023). Metatranscriptomic Analysis Revealed Prevotella as a Potential Biomarker of Oropharyngeal Microbiomes in SARS-CoV-2 Infection. Front. Cell. Infect. Microbiol..

[33] Zhang D., Weng S., Xia C., Ren Y., Liu Z., Xu Y., Yang X., Wu R., Peng L., Sun L. (2023). Gastrointestinal Symptoms of Long COVID-19 Related to the Ectopic Colonization of Specific Bacteria That Move between the Upper and Lower Alimentary Tract and Alterations in Serum Metabolites. BMC Med..

[34] Khan A.A., Khan Z. (2020). COVID-2019-Associated Overexpressed Prevotella Proteins Mediated Host–Pathogen Interactions and Their Role in Coronavirus Outbreak. Bioinformatics.

[35] Soltani S., Faramarzi S., Zandi M., Shahbahrami R., Jafarpour A., Akhavan Rezayat S., Pakzad I., Abdi F., Malekifar P., Pakzad R. (2021). Bacterial Coinfection among Coronavirus Disease 2019 Patient Groups: An Updated Systematic Review and Meta-Analysis. New Microbes New Infect..

[36] Zang C., Zhang Y., Xu J., Bian J., Morozyuk D., Schenck E.J., Khullar D., Nordvig A.S., Shenkman E.A., Rothman R.L. (2023). Data-Driven Analysis to Understand Long COVID Using Electronic Health Records from the RECOVER Initiative. Nat. Commun..

[37] Rovite V., Wolff-Sagi Y., Zaharenko L., Nikitina-Zake L., Grens E., Klovins J. (2018). Genome Database of the Latvian Population (LGDB): Design, Goals, and Primary Results. J. Epidemiol..

