# Supplementary material for: Gut Microbiome Composition and Dynamics in Hospitalized COVID-19 Patients and Patients with Post-Acute COVID-19 Syndrome
Source: Int J Mol Sci. 2024 Jan 1;25(1):567. doi: 10.3390/ijms25010567 (PMC10779053; doi:10.3390/ijms25010567)
Supplement: Supplementary file 1 [file ijms-25-00567-s001.zip › ijms-2761414-supplementary/Supplementary Figures.pdf]

Supplementary Material

Gut microbiome composition and dynamics in hospitalized COVID-19 patients and patients with post-acute COVID-19 syndrome

Monta Brīvība, Laila Silamiķele, Līga Birzniece, Kaspars Megnis, Laura Ansone, Ivars Silamiķelis, Līva Pelcmane, Daniella Borisova, Maija Rozenberga, Lauma Jagare, Ilze Elbere, Jānis Kloviņš.

* Correspondence:
Corresponding Author: monta@biomed.lu.lv

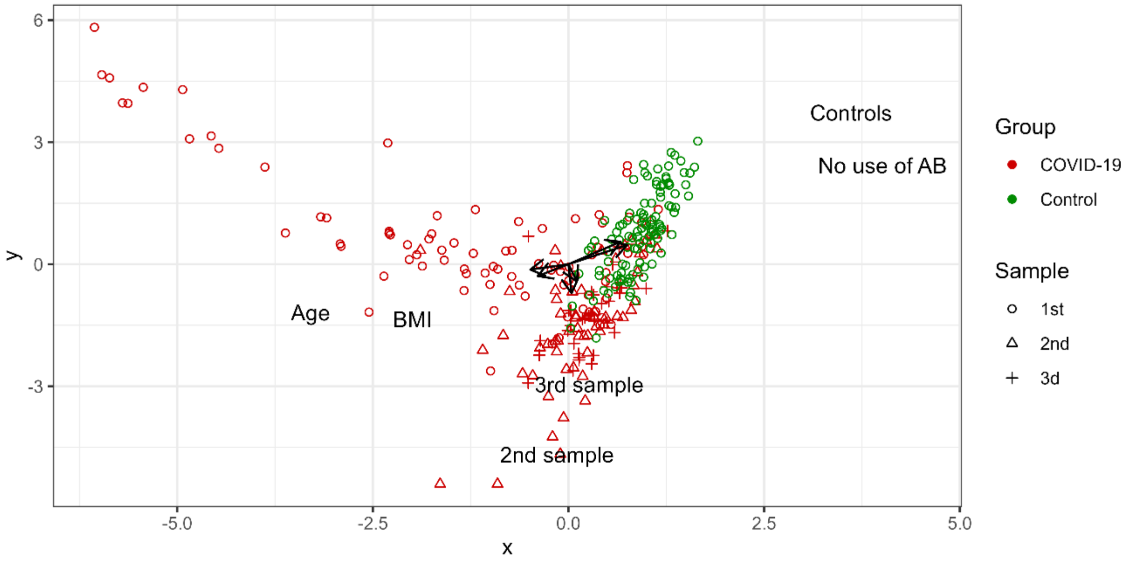

Figure S1. Canonical correspondence analysis (CCA) plot visualizing the correlation of the microbiome between COVID-19 patients (red), healthy controls (green), and sample types. Sample types are as follows: 1st- acute disease phase; 2nd- 1 month since acute phase; 3rd- 3 months since acute disease phase. Environmental factors with significant contributions to the distribution of the gut microbiota community are shown.

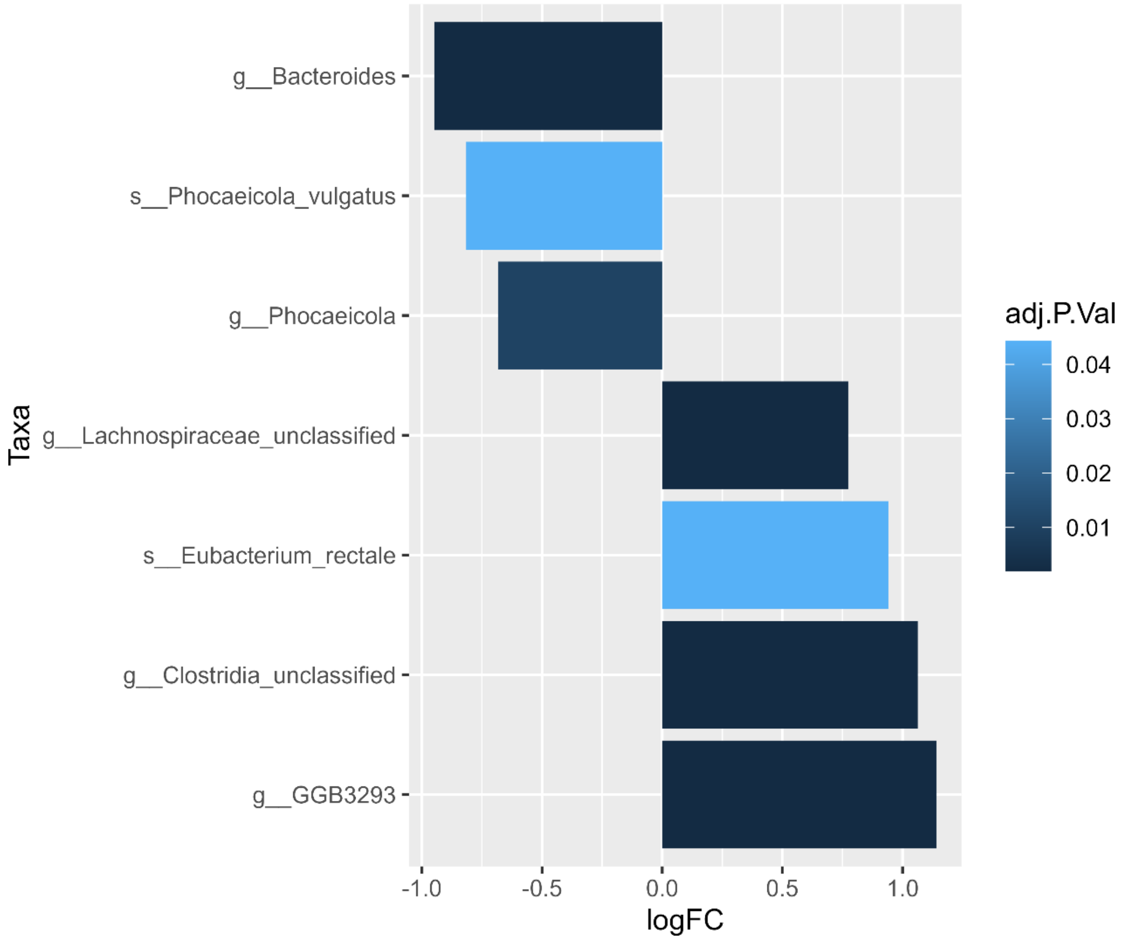

Figure S2. Differential abundance at genus and species levels (expressed as LogFC). Positive LogFC represents taxa with increased abundance in the COVID-19 patient group not administering antibiotics, and negative LogFC – COVID-19 patient group administrating antibiotics.

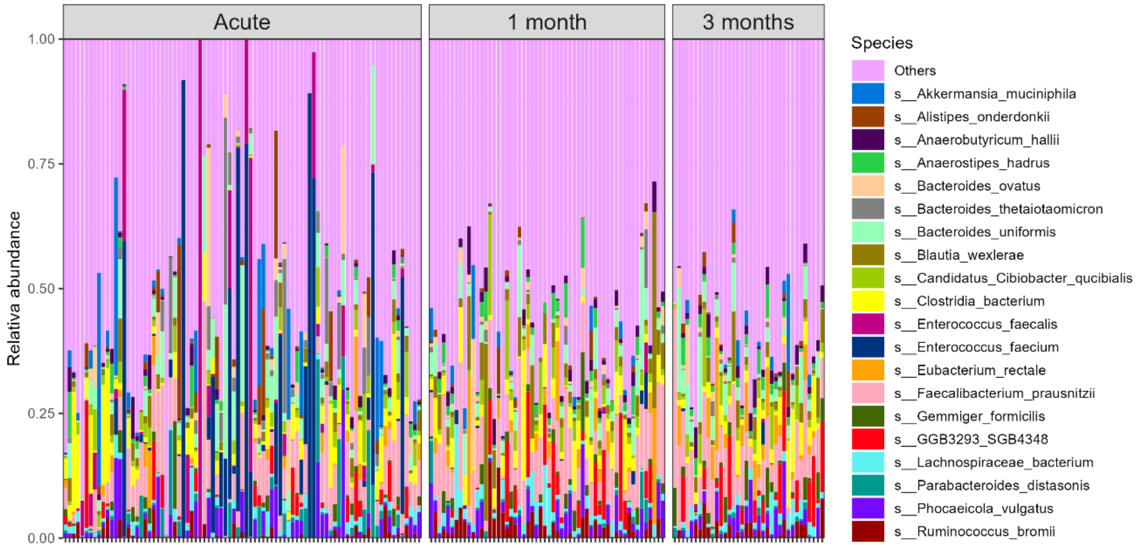

Figure S3. Taxonomy bar plot depicting 20 of the most representative species of samples from hospitalized patients collected at three consecutive time points during the course of the disease.

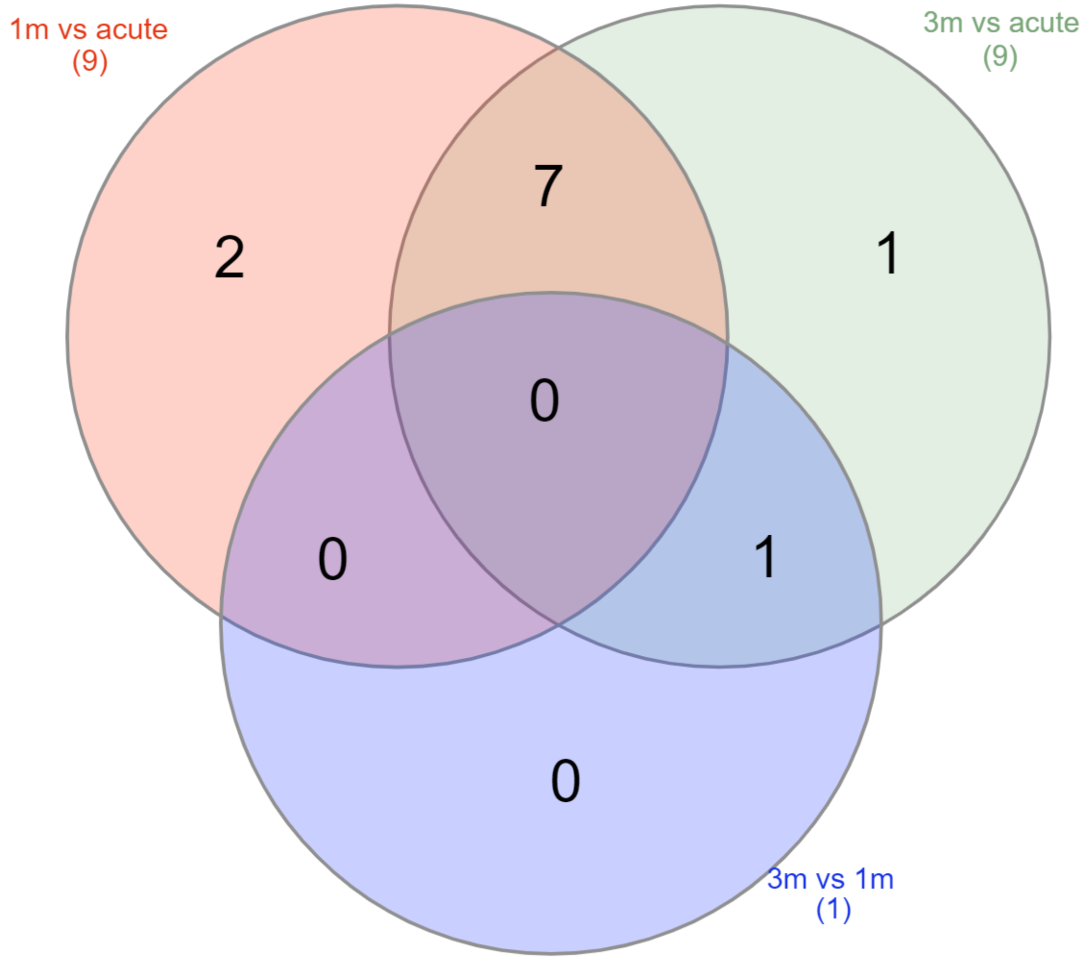

Figure S4. Venn diagram depicting the overlap of significantly altered relative abundance of bacterial species and genus combined among analyzed contrasts. Acute - samples collected during the acute phase of the disease; 1m - samples collected around 1 month since the COVID-19 acute phase; 3m - samples collected around 3 months since the COVID-19 acute phase.
